# Literature Review and Meta-Analysis on Micronutrient Fortified Condiments and Noodles: Reduction of Anemia in Children and Adults

**DOI:** 10.3390/nu8040240

**Published:** 2016-04-23

**Authors:** Olatokunbo Osibogun, Adriana Campa, Purnima Madhivanan

**Affiliations:** Florida International University, Robert Stempel College of Public Health and Social Work, Department of Epidemiology, AHC-5, Miami, FL 33199, USA; campaa@fiu.edu (A.C.); pmadhiva@fiu.edu (P.M.)

Dear Editor,

The importance of micronutrient fortification in countries with limited resources and prevalence of malnutrition cannot be overemphasized. We read with great interest your recent systematic review and meta-analysis [1] published in the February 2016 edition of *Nutrients*. We congratulate the authors for their intent of using two techniques to review the same 14 journal articles. However, it seems that an article’s potential inclusion in the meta-analysis was the basis for the review and not the other way around. This was surprising after the authors had made a great effort in describing the systematic steps to select the articles, which had a set of criteria independent from those used for the meta-analysis. It seems that by unifying the techniques, instead of maintaining them independent from each other, the review was greatly constrained, leading to the long list of limitations described.

The methodology for the systematic review was extensively described in some aspects, but it lacks on others. Firstly, the authors did not report their search strategy in sufficient detail to be reproduced. We performed a search of PubMed using the search strategy provided in Table 1; aside from the difficulty in using what was listed in the table, it did not yield the results that are shown in Figure 1. Secondly, the authors used only one database, it has been found that searching other relevant databases, such as EMBASE, during systematic reviews would decrease bias in addition to identifying other relevant articles [2,3]. Thirdly, the authors say “they also screened an existing literature database”; but failed to mention to which databases they were referring. Without this specific information it is impossible to replicate their review findings. Fourthly, and more importantly, the justification for the exclusion of children under five and adults over 50 years was also not mentioned. These excluded age ranges correspond to populations at high-risk for malnutrition and to potential consumers of the products reviewed in the article. By using these exclusionary age ranges and the condition that only fortified condiments could be included, the authors excluded 819 journal articles (Figure 1). Violating their own expressed rule, however, the authors included one study on fortified noodles [4], which is not a condiment by any measure. Again, this inclusion was probably guided by the structure of this article’s information, which was convenient for a meta-analysis, and not by the steps of a systematic review. The review would have been more focused by using only studies that included condiment and only one technique, in this case the meta-analysis.

Although the systemic review was well described; judging by the results and limitations of this review, it was not well implemented. One of the characteristics of a well-designed and planned systematic review is performing quality and risk of bias assessment of studies, in order to understand the validity of the cumulative evidence reported. The authors carried out appropriate statistical tests; however, they did not give a possible reason for the extreme heterogeneity that was observed in the meta-analysis. It is essential that we know why such heterogeneity exists in order to be able to understand the generalizability of the findings. The authors should have considered subgroup and sensitivity analysis to identify the cause of such heterogeneity and explain those findings. Most likely, the heterogeneity was the product of selection of articles for the meta-analyses and not following the planned steps of a systematic review.

Furthermore, while there is established evidence of the impact of publication bias on systematic reviews and meta-analyses [5], the authors do not report examining for publication bias, which would be one of the indications of a well conducted systematic review and meta-analysis [6]. Interestingly, the authors report funding from a nutrition company. Lesser and colleagues report that research funded by a food industry is more likely to end up favoring such companies [7]. This should be considered when interpreting these results. It should be noted however, that it is difficult to comment on how the methodological shortcomings of this review would impact the findings and conclusions. Finally, the control intervention listed is not quite clear, which further weakens the conclusions from this review. Systematic reviews and meta-analysis are tools, which contribute to helping clinicians and researchers in making decisions, hence the importance for the provision of a high quality review, which will make the findings applicable and valid. It is essential that authors be explicit in the methods used to conduct their systematic reviews, and, therefore, prevent the weaknesses in their conclusions.
